# Association of Haptoglobin Genotype with Cardiovascular Disease and Left Ventricular Ejection Fraction in Kidney Transplant Recipients

**DOI:** 10.3390/jcm15197536

**Published:** 2026-09-28

**Authors:** Zeynep Ural, Ozant Helvacı, Berfu Korucu, Nisa Akın Yetkin, Sevim Gönen, Ulver Derici

**Affiliations:** 1 Department of Nephrology, Lösante Hospital, 06830 Ankara, Turkey; 2 Department of Nephrology, Faculty of Medicine, Gazi University, 06560 Ankara, Turkey; drozant@hotmail.com (O.H.); ulverderici@yahoo.com (U.D.); 3 Department of Nephrology, Faculty of Medicine, Dokuz Eylül University, 35220 Izmir, Turkey; berfukorucu@gmail.com; 4 Sincan Training and Research Hospital, 06949 Ankara, Turkey; drnisaakin@gmail.com; 5 Tissue Typing Laboratory, Faculty of Medicine, Gazi University, 06560 Ankara, Turkey; sevgonen@gmail.com

**Keywords:** haptoglobin genotype, kidney transplantation, cardiovascular disease, left ventricular ejection fraction, echocardiography, cardiometabolic susceptibility

## Abstract

**Background:** Cardiovascular disease remains a major determinant of long-term outcomes after kidney transplantation. Haptoglobin (HP) is a genetically polymorphic hemoglobin-binding protein involved in hemoglobin scavenging, oxidative stress, and inflammation. Although the *HP 2–2* genotype has been associated with cardiometabolic risk in non-transplant populations, its clinical relevance after kidney transplantation remains uncertain. **Methods:** We conducted a single-center retrospective cohort study of 229 adult kidney transplant recipients who underwent recipient *HP* genotyping by polymerase chain reaction. Recipients were categorized a priori as *HP 2–2* (*n* = 119) or non–*HP 2–2* (*HP 2–1*/*HP 1–1*; *n* = 110). Documented cardiovascular disease burden, cardiometabolic characteristics, laboratory parameters, echocardiographic measures, overall biopsy-proven rejection, graft failure, death-censored graft survival, and all-cause mortality were analyzed. Multivariable logistic, linear, and Cox regression models were used as appropriate, with covariates selected according to clinical relevance and event number. **Results:** Compared with non–*HP 2–2* recipients, *HP 2–2* recipients had a higher prevalence of diabetes mellitus (23.5% vs. 12.7%; *p* = 0.03) and documented cardiovascular disease burden (24.4% vs. 10.9%; *p* = 0.008). Median left ventricular ejection fraction (LVEF) was lower in *HP 2–2* recipients (65% [interquartile range (IQR), 60–67] vs. 68% [IQR, 62–70]; *p* < 0.001), with higher unadjusted frequencies of sex-defined left atrial enlargement and left ventricular hypertrophy. In multivariable analyses, *HP 2–2* showed nominal associations with documented cardiovascular disease burden (adjusted odds ratio (aOR), 2.32; 95% confidence interval (CI), 1.07–5.03; *p* = 0.033; false discovery rate (FDR) q = 0.163) and lower continuous LVEF (β, −2.00 percentage points; 95% CI, −3.61 to −0.38; *p* = 0.015; FDR q = 0.154); neither association remained significant after FDR correction. In complementary allele-dosage analyses, increasing *HP2* allele dosage remained associated with lower LVEF after FDR correction (β, −1.93 percentage points per additional *HP2* allele; 95% CI, −3.20 to −0.65; *p* = 0.0033; FDR q = 0.036). Overall biopsy-proven rejection, graft failure, death-censored graft survival, and all-cause mortality were not associated with *HP 2–2*. **Conclusions:** Recipient *HP 2–2* genotype showed nominal associations with greater documented cardiovascular disease burden and lower LVEF, whereas the graded inverse association between *HP2* allele dosage and LVEF remained after FDR correction. These findings are exploratory and require prospective validation before clinical risk-stratification implications can be considered.

## 1. Introduction

Kidney transplantation is the preferred treatment for end-stage kidney disease, providing a survival advantage and improved quality of life compared with long-term dialysis [1]. Despite these benefits, long-term outcomes after kidney transplantation remain strongly influenced by cardiovascular disease, which continues to be a major cause of morbidity and mortality in kidney transplant recipients [2,3,4]. Conventional cardiovascular risk factors, including diabetes mellitus, hypertension, dyslipidemia, reduced kidney function, and prior cardiovascular disease, are highly prevalent after transplantation, but they do not fully explain the substantial interindividual variability in cardiovascular outcomes [2,3,4,5]. This residual heterogeneity suggests that biologically plausible modifiers of oxidative stress, inflammation, and vascular injury may contribute to cardiovascular susceptibility in selected transplant recipients.

Haptoglobin is a circulating hemoglobin-binding protein with antioxidant, anti-inflammatory, and hemoglobin-scavenging functions [6,7,8]. The *HP* gene is structurally polymorphic and gives rise to three common genotypes: *HP 1–1*, *HP 2–1*, and *HP 2–2* [6,9]. These genotypes encode haptoglobin proteins that differ in polymeric structure, hemoglobin-binding complex handling, antioxidant capacity, and downstream inflammatory signaling [8,10,11,12]. In particular, *HP 2–2* has been associated with less efficient clearance of hemoglobin–iron complexes, increased redox-active iron exposure, impaired antioxidant protection, and greater oxidative stress [8,10,11,12]. A conceptual overview of the genotype-dependent differences in haptoglobin structure, hemoglobin-scavenging efficiency, CD163-mediated clearance, and downstream oxidative-inflammatory consequences is provided in Supplementary Figure S2. Human plaque studies further support this biological framework by showing that *HP 2–2* is associated with increased plaque iron deposition, oxidation-specific epitopes, apoptosis, impaired hemoglobin clearance, and enhanced inflammatory and angiogenic responses in atherosclerotic tissue [13,14].

The cardiovascular relevance of *HP* genotype appears to be context dependent, with the strongest evidence arising from diabetic and hyperglycemic populations. In prospective and trial-based cohorts, *HP 2–2* has been linked to higher cardiovascular risk particularly in the presence of diabetes mellitus, elevated HbA1c, or adverse metabolic exposure [15,16,17,18,19,20]. Contemporary analyses using repeated or time-varying HbA1c measurements have further suggested that the association between glycemic exposure and coronary outcomes may differ according to *HP* genotype [21,22,23]. These observations support a model in which *HP 2–2* does not act as a uniform cardiovascular risk genotype across all individuals, but rather may confer susceptibility under conditions of metabolic and oxidative stress.

Kidney transplant recipients provide a particularly informative population in which to investigate this context-dependent susceptibility. Unlike the general population, and distinct from other solid-organ transplant populations that do not uniformly have a preceding history of advanced chronic kidney disease or kidney failure, kidney transplant recipients enter transplantation with accumulated cardiorenal and vascular injury related to prolonged kidney dysfunction, uremic exposure, and, in many patients, dialysis. Although kidney transplantation markedly improves survival, it does not normalize cardiovascular risk. In a contemporary nationwide cohort, kidney transplant recipients had substantially higher 5-year risks of myocardial infarction, stroke, or heart failure and all-cause mortality than age- and sex-matched non-transplant controls [24]. Residual allograft dysfunction, persistent hypertension, chronic immunosuppressive exposure, and transplant-associated metabolic disturbances further sustain this cardiovascular-risk environment. Dysglycemia is particularly relevant: post-transplant glucose abnormalities are common and increasing post-transplant glycemic exposure has been associated with cardiovascular events [25], while randomized data demonstrate that calcineurin-inhibitor choice can modify post-transplant glucose metabolism [26]. Thus, kidney transplant recipients combine a pre-existing kidney disease–related vascular substrate with persistent cardiorenal and treatment-related metabolic stress, providing a biologically plausible setting in which genotype-dependent differences in hemoglobin scavenging and antioxidant efficiency may contribute to cardiovascular susceptibility.

Against this biological background, the clinical relevance of *HP* genotype in transplantation remains incompletely defined and may vary according to organ type and clinical context. Donor or recipient haptoglobin variation has been associated with outcomes in liver transplantation, cardiac allograft vasculopathy after heart transplantation, and inflammatory complications after hematopoietic cell transplantation [27,28,29], supporting the possibility that the phenotypic expression of HP-related biology is influenced by the surrounding metabolic, vascular, and inflammatory environment. Kidney transplant–specific evidence, however, remains limited. In a large cohort of kidney transplant recipients, *HP 2–2* was not associated with cardiovascular or all-cause mortality, overall graft loss, or death-censored graft loss [30]. Importantly, these late, multifactorial outcomes may not capture intermediate cardiovascular phenotypes that are more proximal to oxidative stress, vascular injury, and cardiac functional alterations. Whether recipient *HP* genotype is associated with documented cardiovascular disease burden or echocardiographic cardiac phenotypes after kidney transplantation therefore remains insufficiently characterized.

On this basis, we hypothesized that recipient *HP 2–2* genotype would be associated with greater documented cardiovascular phenotype burden and adverse echocardiographic characteristics, particularly lower LVEF, while its relationship with hard transplant-related outcomes might be less apparent. Accordingly, we investigated associations of recipient *HP* genotype with cardiometabolic characteristics, documented cardiovascular disease burden, echocardiographic measures, and transplant-related outcomes in a single-center cohort of adult kidney transplant recipients. The a priori primary comparison was *HP 2–2* versus non–*HP 2–2*, complemented by three-genotype and *HP2* allele-dosage sensitivity analyses.

## 2. Materials and Methods

### 2.1. Study Design and Population

This was a retrospective, single-center cohort study of adult kidney transplant recipients followed at the transplant outpatient clinic of the Department of Nephrology, Gazi University Faculty of Medicine. Adult kidney transplant recipients who were already under routine follow-up at the transplantation outpatient clinic as of the ethics approval date were retrospectively screened for eligibility using pre-existing clinical records. The primary outcomes were documented cardiovascular disease burden and continuous left ventricular ejection fraction. Secondary outcomes included diabetes mellitus, other echocardiographic parameters, overall biopsy-proven rejection, progression to ESKD/graft failure, death-censored graft survival, and all-cause mortality. The study was conducted in accordance with the Declaration of Helsinki and was approved by the Gazi University Clinical Research Ethics Committee (approval no. 289; date of approval: 22 March 2021). Written informed consent was obtained from all participants before inclusion.

### 2.2. Patient Selection

A total of 298 adult kidney transplant recipients were screened. Forty-five recipients were excluded before formation of the genotype-eligible cohort because of inactive or unavailable longitudinal follow-up (*n* = 30) or incomplete core clinical outcome data (*n* = 15). Among 253 eligible recipients with analyzable clinical data, recipient *HP* genotyping was successfully completed in 229. Genotyping was not completed in 24 recipients because of DNA/sample integrity failure (*n* = 4) or unavailable or insufficient blood/DNA samples (*n* = 20). The final analytic cohort therefore included 229 kidney transplant recipients with successful recipient *HP* genotyping (Figure 1).

### 2.3. Haptoglobin Genotyping

Recipient *HP* genotype was determined using a GAP-PCR–based genotyping protocol. Genomic DNA was extracted using the EZ1 Advanced system (QIAGEN GmbH, Hilden, Germany). Three primers (HP_2F, HP_GAP_F, and HP_6R) were used to distinguish the *HP1* and *HP2* alleles. PCR products were resolved by 2% agarose gel electrophoresis and visualized under ultraviolet illumination. Genotypes were assigned according to the resulting band pattern: a single 2050-bp product indicated *HP 1–1*, a single 1769-bp product indicated *HP 2–2*, and the presence of both products indicated *HP 2–1*. Full primer sequences and the detailed GAP-PCR design, including the theoretical 3769-bp *HP2*-related product, are provided in Supplementary File S1 and Supplementary Figure S1. 

Because the exposure of interest was determined by DNA-based testing, results are reported as recipient *HP* genotype throughout the manuscript. Recipients were categorized a priori as *HP 2–2* or non–*HP 2–2*, with the latter group comprising *HP 2–1* and *HP 1–1* genotypes. This grouping was selected because *HP 2–2* has been evaluated as the principal susceptibility genotype in prior cardiometabolic and diabetic cardiovascular studies and because the *HP 1–1* subgroup was small in the present cohort. The genotyping call rate was calculated among genotype-eligible recipients, and genotype distribution was assessed for Hardy–Weinberg equilibrium. Three-genotype comparisons and *HP2* allele-dosage analyses were performed as sensitivity analyses. Circulating serum or plasma haptoglobin concentration was not measured as part of the study protocol; therefore, the present analyses specifically evaluate genetically determined recipient *HP* genotype rather than circulating haptoglobin levels.

### 2.4. Clinical Data Collection

Demographic, clinical, laboratory, transplant-related, echocardiographic, and outcome data were retrospectively abstracted from institutional electronic medical records. Baseline and transplant-related variables included age, sex, donor type, dialysis modality before transplantation, primary cause of kidney failure, calcineurin inhibitor use, mechanistic target of rapamycin inhibitor use, antimetabolite use, and maintenance steroid dose. Dialysis modality before transplantation was categorized as preemptive transplantation, hemodialysis, peritoneal dialysis, or combined hemodialysis/peritoneal dialysis or other modality according to registry coding. Primary causes of kidney failure were grouped as glomerulonephritis/immunologic diseases, diabetic nephropathy, and other causes.

Cardiometabolic phenotypes were analyzed as cumulative documented disease burden recorded at any time before transplantation or during post-transplant follow-up. These variables were therefore not treated as uniformly ascertained baseline exposures or as adjudicated incident outcomes. Cardiovascular disease was defined as documented coronary artery disease, prior myocardial infarction, coronary revascularization, ischemic stroke, or peripheral artery disease. Diabetes mellitus included both pre-existing and post-transplant disease and was defined as a documented diagnosis before or after transplantation, current antidiabetic medication use, fasting plasma glucose ≥ 126 mg/dL on two separate occasions, or hemoglobin A1c ≥ 6.5%. Malignancy was defined as any solid-organ or hematologic malignancy diagnosed during follow-up. Cytomegalovirus infection and BK viremia were recorded when documented in the medical record by clinical diagnosis and/or virologic testing.

Laboratory data were obtained from the most recent available follow-up assessment and included blood urea nitrogen, serum creatinine, fasting plasma glucose, LDL cholesterol, triglycerides, and total cholesterol. Laboratory values were reported in US conventional units.

### 2.5. Rejection and Transplant Outcome Definitions

Biopsy-proven rejection was defined as any histologically confirmed kidney allograft rejection episode recorded in the pathology report and classified according to Banff criteria in use at the time of biopsy. Because donor-specific antibody data, mean fluorescence intensity thresholds, HLA mismatch data, Banff version standardization, and internally concordant subtype-level coding were not uniformly available across the retrospective cohort, rejection was analyzed only as overall biopsy-proven rejection.

Progression to end-stage kidney disease/graft failure was defined as irreversible graft failure requiring permanent return to dialysis or retransplantation. Death-censored graft survival was defined as the interval from kidney transplantation to graft failure, with death with a functioning graft treated as a censoring event. Recipients who remained alive with a functioning graft were censored at the last documented follow-up visit. Death-censored graft survival was analyzed over the full available follow-up period. No fixed 5-year surveillance window was imposed in the primary analysis because follow-up duration extended beyond 5 years in a substantial proportion of recipients. All-cause mortality was defined as death from any cause during follow-up and was analyzed as a binary outcome because time-to-death data were not uniformly available.

### 2.6. Echocardiographic Assessment

Transthoracic echocardiographic data were collected from routine post-transplant clinical assessments. Continuous LVEF was available for all 229 recipients. Structural echocardiographic variables used for left atrial and left ventricular structural analyses were available in 191 recipients (83.4%) and were analyzed using an available-case approach without imputation. Recipients without evaluable structural echocardiographic data remained included in all other analyses. Structural echocardiographic availability was defined by availability of left atrial diameter and left ventricular hypertrophy data. When more than one echocardiographic examination was available, the most recent examination before last follow-up, graft failure, or death was used. Because echocardiography was obtained during routine clinical care rather than according to a protocolized study schedule, differential referral or ascertainment could not be excluded. Importantly, the selected echocardiographic examinations were not performed at a uniform time point after kidney transplantation. The exact transplant-to-echocardiography interval was not reliably available in the analytic dataset and therefore could not be summarized or incorporated as a covariate. Accordingly, the echocardiographic findings represent routine-care cardiac phenotypes assessed at heterogeneous post-transplant time points, with potential for temporal confounding.

Left ventricular ejection fraction was analyzed primarily as a continuous variable. A secondary categorical variable for left ventricular ejection fraction < 60% was recalculated directly from the recorded left ventricular ejection fraction value; the original database-derived binary variable for reduced ejection fraction was not used. Left atrial diameter was analyzed as a continuous echocardiographic measure. Left atrial enlargement was defined using sex-specific anteroposterior left atrial diameter thresholds: ≥3.8 cm in women and ≥4.0 cm in men. Left ventricular hypertrophy was recorded according to the echocardiographic report. Echocardiographic measurements were interpreted by experienced cardiologists as part of routine clinical care.

### 2.7. Statistical Analysis

Continuous variables were summarized as median with interquartile range. Categorical variables were summarized as counts and percentages. The primary exposure was recipient *HP 2–2* genotype. The primary comparison was *HP 2–2* versus non–*HP 2–2*. Between-group comparisons were performed using the Mann–Whitney U test for continuous variables. Categorical variables were compared using Pearson chi-square tests when expected cell counts were adequate; Fisher’s exact test or the Fisher–Freeman–Halton exact test was used for sparse two-category or multi-category comparisons, respectively. All statistical tests were two-sided.

Clinical outcomes of interest included cardiometabolic phenotypes, documented cardiovascular disease burden, echocardiographic measures, overall biopsy-proven rejection, progression to end-stage kidney disease/graft failure, death-censored graft survival, and all-cause mortality. Multivariable logistic regression was used for binary outcomes, linear regression for continuous echocardiographic outcomes, and Cox proportional hazards regression for death-censored graft failure. Covariates were selected a priori based on clinical relevance and limited according to the number of events to reduce overfitting.

For documented cardiovascular disease burden, the adjusted logistic regression model included *HP 2–2* genotype, age, sex, diabetes mellitus, and hypertension. For diabetes mellitus, the adjusted model included *HP 2–2* genotype, age, and sex. For continuous left ventricular ejection fraction, left atrial diameter, left ventricular hypertrophy, left atrial enlargement, and left ventricular ejection fraction < 60%, adjusted models included *HP 2–2* genotype, age, sex, diabetes mellitus, and hypertension. For overall biopsy-proven rejection and progression to end-stage kidney disease/graft failure, adjusted logistic regression models included *HP 2–2* genotype, age, sex, and donor type. For all-cause mortality, the adjusted logistic regression model included *HP 2–2* genotype, age, and sex because of the limited number of events. Death-censored graft survival was assessed using the Kaplan–Meier method, and survival distributions were compared using the log-rank test. Cox proportional hazards regression for death-censored graft failure included *HP 2–2* genotype, age, sex, and donor type. The proportional hazards assumption was assessed using standard diagnostic methods. Because sex may influence echocardiographic measurements, sex was retained a priori in all multivariable echocardiographic models irrespective of its unadjusted distribution between genotype groups.

Sensitivity analyses included three-genotype comparisons among *HP 1–1*, *HP 2–1*, and *HP 2–2* recipients and *HP2* allele-dosage models coded as *HP 1–1* = 0, *HP 2–1* = 1, and *HP 2–2* = 2. Because the *HP 1–1* subgroup was small, three-genotype comparisons were considered descriptive. Allele-dosage models used the same outcome-specific covariate sets as the primary analyses.

To characterize potential selection associated with structural echocardiographic availability, recipients with and without available structural data were compared in a post hoc missingness analysis (Supplementary Table S3). Absolute standardized differences (SMDs) were used as the primary balance metric, with *p* values provided as secondary descriptive measures. Continuous variables were compared using the Mann–Whitney U test, binary categorical variables using Fisher’s exact test, and multicategory variables using the Fisher–Freeman–Halton exact test. An absolute SMD ≥ 0.10 was considered indicative of potentially meaningful imbalance for descriptive interpretation. No multiplicity correction was applied to this descriptive missingness assessment. To further evaluate potential residual confounding, post hoc sensitivity analyses were performed using additional transplant-related covariates reliably available in the final dataset (Supplementary Table S4). Starting from the prespecified adjustment set of age, sex, diabetes mellitus, and hypertension, most recent serum creatinine, graft follow-up duration, donor type, pre-transplant dialysis modality, calcineurin inhibitor regimen, mTOR inhibitor use, antimetabolite use, and maintenance steroid category were added individually. A core transplant-adjusted model including serum creatinine, graft follow-up duration, and donor type and an exploratory fully expanded available-covariate model additionally incorporating maintenance immunosuppressive exposures were evaluated. Because documented cardiovascular disease was present in only 41 recipients, extensively parameterized cardiovascular disease models were considered exploratory. For continuous LVEF, an additional model further adjusted for documented cardiovascular disease burden was examined. A separately adjudicated baseline-only cardiovascular disease variable, the exact transplant-to-echocardiography interval, validated dialysis duration, standardized longitudinal anemia/hemoglobin data, smoking status, and lipid-lowering treatment were not reliably available and therefore could not be incorporated.

Analyses were conducted using available data for each outcome, and outcome-specific denominators are reported where applicable. Descriptive comparisons used the Mann–Whitney U test for continuous variables and Pearson’s chi-square test or Fisher’s exact test for categorical variables, as appropriate. False discovery rate correction was performed using the Benjamini–Hochberg procedure separately within the two analytic frameworks. For the *HP 2–2* versus non–*HP 2–2* analyses, correction was applied across the 10 adjusted outcome models included in the primary adjusted model family; the descriptive graft follow-up-duration comparison was not included in this model family. For the complementary *HP2* allele-dosage analyses, correction was applied across the 11 adjusted models reported in Supplementary Table S2. Exact model-derived *p* values were used for FDR correction before rounding. Findings with nominal *p* < 0.05 but FDR q ≥ 0.05 were considered nominal and hypothesis-generating. *p* values for the nested residual-confounding sensitivity models were considered exploratory and were not subjected to an additional FDR correction because these models assessed robustness of the same underlying associations rather than independent hypotheses. All tests were two-sided, and statistical analyses were performed using SPSS Statistics version 25.0 (IBM Corp., Armonk, NY, USA).

## 3. Results

### 3.1. Study Cohort and Genotype Distribution

A total of 298 adult kidney transplant recipients who were already under routine follow-up at the transplantation outpatient clinic were retrospectively screened for eligibility. Forty-five recipients were excluded before eligibility assessment because of inactive or unavailable follow-up (*n* = 30) or incomplete core clinical data (*n* = 15). Among 253 eligible recipients, recipient *HP* genotyping was successfully completed in 229, yielding a genotyping call rate of 90.5%. The final analytic cohort therefore included 229 kidney transplant recipients with successful *HP* genotyping (Figure 1).

The *HP 2–2* genotype was present in 119 recipients (52.0%). The non–*HP 2–2* group included 110 recipients, comprising 93 with *HP 2–1* and 17 with *HP 1–1* genotypes. Genotype distribution did not deviate from Hardy–Weinberg equilibrium (*p* = 0.84).

### 3.2. Baseline and Transplant-Related Characteristics

Baseline and transplant-related characteristics according to *HP* genotype are shown in Table 1. Median age was 41 years (IQR, 32–50) in the overall cohort and did not differ significantly between *HP 2–2* and non–*HP 2–2* recipients (41 [IQR, 34–50] vs. 38 [IQR, 29–50] years; *p* = 0.13). Female sex was similarly distributed between groups (33.6% vs. 37.3%; *p* = 0.56).

Living donor transplantation was numerically less frequent among *HP 2–2* recipients than among non–*HP 2–2* recipients, but this difference did not reach statistical significance (71.4% vs. 81.8%; *p* = 0.06). Dialysis modality before transplantation, primary cause of kidney failure, calcineurin inhibitor use, mTOR inhibitor use, antimetabolite use, and maintenance steroid dose were not significantly different between genotype groups (Table 1).

### 3.3. Cardiometabolic and Laboratory Phenotypes

Cardiometabolic, infection-related, laboratory, and echocardiographic characteristics are summarized in Table 2. Hypertension was common in both groups and did not differ according to *HP* genotype. Diabetes mellitus was documented in 42/229 recipients (18.3%) and was more frequent among *HP 2–2* than non–*HP 2–2* recipients (28/119 [23.5%] vs. 14/110 [12.7%]; *p* = 0.03). Documented cardiovascular disease burden was also greater in the *HP 2–2* group (29/119 [24.4%] vs. 12/110 [10.9%]; *p* = 0.008). These variables represent cumulative documented clinical phenotypes recorded before transplantation or during post-transplant follow-up and should not be interpreted as baseline-only or incident outcomes.

Post-transplant infection events were also similar between genotype groups. CMV infection occurred in 16 *HP 2–2* recipients and 13 non–*HP 2–2* recipients (13.4% vs. 11.8%; *p* = 0.71), and BK viremia occurred in 6 *HP 2–2* recipients and 4 non–*HP 2–2* recipients (5.0% vs. 3.6%; Fisher exact *p* = 0.75). Laboratory measures at last follow-up were broadly similar between genotype groups. Blood urea nitrogen, serum creatinine, fasting plasma glucose, LDL cholesterol, triglycerides, and total cholesterol did not differ significantly between *HP 2–2* and non–*HP 2–2* recipients (Table 2).

### 3.4. Echocardiographic Phenotypes

Echocardiographic findings according to *HP* genotype are presented in Table 2 and Figure 2. Continuous LVEF was available for all 229 recipients, whereas structural echocardiographic data were available in 191 recipients (83.4%). Structural data were available in 107/119 *HP 2–2* recipients (89.9%) and 84/110 non–*HP 2–2* recipients (76.4%; Fisher exact *p* = 0.007). In the dedicated availability analysis (Supplementary Table S3), meaningful imbalances were observed for *HP 2–2* genotype (|SMD| = 0.508), documented cardiovascular disease burden (|SMD| = 0.352), age (|SMD| = 0.290), diabetes mellitus (|SMD| = 0.263), and selected transplant- and immunosuppression-related characteristics. These findings indicate that differential ascertainment of structural echocardiographic data cannot be excluded. Median LVEF was lower in *HP 2–2* recipients than in non–*HP 2–2* recipients (65% [IQR, 60–67] vs. 68% [IQR, 62–70]; *p* < 0.001).

Among recipients with available structural data, left atrial diameter was numerically higher in the *HP 2–2* group (3.7 [IQR, 3.3–4.0] vs. 3.5 [IQR, 3.3–3.8] cm; *p* = 0.06). Sex-defined left atrial enlargement was more frequent among *HP 2–2* recipients (41/107 [38.3%] vs. 20/84 [23.8%]; *p* = 0.03), as was left ventricular hypertrophy (43/107 [40.2%] vs. 17/84 [20.2%]; *p* = 0.003). Because structural echocardiographic availability was non-uniform, these unadjusted structural findings are considered secondary and exploratory.

In adjusted analyses, *HP 2–2* genotype showed nominal associations with documented cardiovascular disease burden (aOR, 2.32; 95% CI, 1.07–5.03; *p* = 0.033; FDR q = 0.163) and lower continuous LVEF (β, −2.00 percentage points; 95% CI, −3.61 to −0.38; *p* = 0.015; FDR q = 0.154). Neither association remained statistically significant after FDR correction. *HP 2–2* was not independently associated with LVEF < 60% (aOR, 1.89; 95% CI, 0.86–4.18; *p* = 0.114; FDR q = 0.284), left atrial diameter (β, 0.06 cm; 95% CI, −0.05 to 0.18; *p* = 0.279; FDR q = 0.465), sex-defined left atrial enlargement (aOR, 1.58; 95% CI, 0.79–3.17; *p* = 0.197; FDR q = 0.393), or left ventricular hypertrophy (aOR, 2.10; 95% CI, 1.00–4.42; *p* = 0.051; FDR q = 0.171).

Additional multivariable sensitivity analyses are presented in Supplementary Table S4. When serum creatinine, graft follow-up duration, donor type, pre-transplant dialysis modality, calcineurin inhibitor regimen, mTOR inhibitor use, antimetabolite use, or maintenance steroid category were individually added to the prespecified adjustment set, the *HP 2–2* effect estimate for documented cardiovascular disease remained directionally similar, with aORs ranging from 2.12 to 2.55. In the core transplant-adjusted model including serum creatinine, graft follow-up duration, and donor type, the association was attenuated (aOR, 2.20; 95% CI, 0.98–4.96; *p* = 0.057); in the fully expanded available-covariate model, the estimate remained similar in magnitude but was less precise (aOR, 2.14; 95% CI, 0.94–4.86; *p* = 0.069). The continuous LVEF association was comparatively stable across individual covariate sensitivity models (β range, −1.85 to −2.14 percentage points) and persisted in the fully expanded model (β, −2.00; 95% CI, −3.65 to −0.36; *p* = 0.017). Further adjustment for documented cardiovascular disease burden yielded an attenuated but directionally consistent estimate (β, −1.72; 95% CI, −3.36 to −0.09; *p* = 0.039). The complete set of adjusted cardiovascular, echocardiographic, and transplant-related associations is summarized in Table 3.

### 3.5. Transplant-Related Outcomes and Death-Censored Graft Survival

Transplant-related outcomes are also shown in Table 3. Overall biopsy-proven rejection occurred in 31 recipients (13.5%) and did not differ significantly between *HP 2–2* and non–*HP 2–2* recipients (11.8% vs. 15.5%; *p* = 0.41). In adjusted logistic regression including age, sex, and donor type, *HP 2–2* was not associated with overall biopsy-proven rejection (aOR, 0.80; 95% CI, 0.37–1.74; *p* = 0.581; FDR q = 0.685).

Progression to ESKD/graft failure occurred in 31 recipients (13.5%) and was similar between *HP 2–2* and non–*HP 2–2* groups (15.1% vs. 11.8%; *p* = 0.46). In adjusted logistic regression including age, sex, and donor type, *HP 2–2* was not associated with progression to ESKD/graft failure (aOR, 1.27; 95% CI, 0.59–2.76; *p* = 0.543; FDR q = 0.685).

Median death-censored graft follow-up duration was 108 months (IQR, 72–168) in the overall cohort, with no significant difference between *HP 2–2* and non–*HP 2–2* recipients (120 [IQR, 78–168] vs. 102 [IQR, 63–156] months; *p* = 0.15). Kaplan–Meier analysis showed no significant difference in death-censored graft survival (log-rank p = 0.59; Figure 3). In Cox proportional hazards regression adjusted for age, sex, and donor type, *HP 2–2* was not associated with death-censored graft failure (aHR, 1.16; 95% CI, 0.56–2.41; *p* = 0.685; FDR q = 0.685).

All-cause mortality occurred in 24 recipients (10.5%) and did not differ between *HP 2–2* and non–*HP 2–2* recipients (10.1% vs. 10.9%; *p* = 0.84). In adjusted logistic regression including age and sex, *HP 2–2* was not associated with all-cause mortality (aOR, 0.83; 95% CI, 0.33–2.05; *p* = 0.682; FDR q = 0.685).

### 3.6. Three-Genotype and HP2 Allele-Dosage Sensitivity Analyses

Three-genotype comparisons are presented in Supplementary Table S1. Because the *HP 1–1* subgroup was small (*n* = 17), these analyses were considered descriptive. Diabetes mellitus, documented cardiovascular disease burden, left ventricular ejection fraction, and left ventricular hypertrophy differed across the three genotype groups. Documented cardiovascular disease was observed in 5.9% of *HP 1–1* recipients, 11.8% of *HP 2–1* recipients, and 24.4% of *HP 2–2* recipients (*p* = 0.02). Median left ventricular ejection fraction was highest in *HP 1–1* recipients and lowest in *HP 2–2* recipients (70% [IQR, 67–70], 67% [IQR, 62–70], and 65% [IQR, 60–67], respectively; *p* < 0.001). Overall biopsy-proven rejection, progression to ESKD/graft failure, all-cause mortality, and death-censored graft follow-up duration did not differ significantly across the three genotype groups.

Adjusted *HP2* allele-dosage models are shown in Supplementary Table S2. Increasing *HP2* allele dosage was associated with lower LVEF after adjustment for age, sex, diabetes mellitus, and hypertension (β, −1.93 percentage points per additional *HP2* allele; 95% CI, −3.20 to −0.65; *p* = 0.0033; FDR q = 0.036). Associations between *HP2* allele dosage and diabetes mellitus (aOR, 2.15; 95% CI, 1.15–4.03; *p* = 0.017; FDR q = 0.095) and documented cardiovascular disease burden (aOR, 2.10; 95% CI, 1.05–4.18; *p* = 0.035; FDR q = 0.130) were nominal and did not remain significant after FDR correction. *HP2* allele dosage was not associated with overall biopsy-proven rejection, progression to ESKD/graft failure, death-censored graft failure, or all-cause mortality. The inverse LVEF association was robust to broader adjustment: in the fully expanded available-covariate model, each additional *HP2* allele was associated with a 1.83-percentage-point lower LVEF (95% CI, −3.12 to −0.53; *p* = 0.006), and after further adjustment for documented cardiovascular disease burden the estimate remained −1.60 percentage points per *HP2* allele (95% CI, −2.89 to −0.31; *p* = 0.015).

## 4. Discussion

In this single-center cohort of adult kidney transplant recipients, recipient *HP 2–2* genotype showed nominal associations with greater documented cardiovascular disease burden and lower continuous LVEF, but neither association remained significant after FDR correction. In complementary *HP2* allele-dosage analyses, increasing *HP2* allele burden showed a graded inverse association with LVEF that remained after FDR correction. Higher unadjusted frequencies of left atrial enlargement and left ventricular hypertrophy were also observed among *HP 2–2* recipients, whereas overall biopsy-proven rejection, progression to ESKD/graft failure, death-censored graft survival, and all-cause mortality were not associated with *HP 2–2*. Collectively, these findings support an exploratory intermediate cardiovascular phenotype signal rather than independent cardiovascular risk prediction.

Cardiovascular disease remains a central determinant of long-term morbidity and mortality after kidney transplantation despite the survival advantage of transplantation over dialysis [1,2,3,4]. Conventional risk factors, including diabetes mellitus, hypertension, dyslipidemia, reduced kidney function, and prior cardiovascular disease, do not fully explain the heterogeneity of cardiovascular outcomes in kidney transplant recipients [2,3,4,5]. The present study therefore evaluated a biologically plausible oxidative-stress-related genotype in relation to cardiovascular and echocardiographic phenotypes. Cardiovascular disease in this cohort should be understood as cumulative documented disease burden rather than a prospectively adjudicated incident endpoint.

The biological plausibility of the observed association is supported by known structure–function differences among haptoglobin genotypes. Haptoglobin binds free hemoglobin and contributes to hemoglobin scavenging, antioxidant defense, macrophage signaling, and modulation of inflammation [6,7,8,11,12,31,32]. Compared with *HP 1–1*, *HP 2–2* encodes a larger polymeric protein with less efficient hemoglobin–iron complex handling and reduced antioxidant capacity, which may increase redox-active iron exposure, oxidative stress, and vascular inflammation [8,10,11,12]. Human plaque studies provide particularly relevant mechanistic support. In diabetic atherosclerotic tissue, *HP 2–2* has been associated with impaired hemoglobin clearance, increased intraplaque iron deposition, oxidative stress, macrophage-rich inflammation, and angiogenic plaque features [13]. In human aortic plaques, *HP 2–2* has also been linked to increased oxidation-specific epitopes and apoptosis, suggesting genotype-dependent vulnerability to oxidative plaque injury [14]. These mechanisms are relevant to kidney transplant recipients, in whom metabolic stress, chronic inflammation, reduced kidney function, immunosuppressive exposure, and vascular disease frequently coexist.

The cardiovascular relevance of *HP* genotype appears to be context dependent, with the strongest evidence arising from diabetic and hyperglycemic populations. Earlier observational and type 1 diabetes studies suggested that adverse cardiovascular effects associated with the *HP2* allele become more apparent in the presence of diabetes or poor glycemic control [15,16,17,18,33,34]. Trial-based analyses have further refined this concept. In ACCORD, intensive glucose lowering was associated with lower coronary heart disease risk among participants with the *Hp 2-2* phenotype, while analyses incorporating longitudinal HbA1c supported a relationship between sustained glycemic exposure and coronary risk [19,21]. In contrast, Look AHEAD did not consistently reproduce these associations [22], and in ADVANCE, intensive glycemic control was not associated with a significant reduction in coronary events across the overall *Hp 2-2* group, although a benefit was observed among participants without cardiovascular disease at baseline [35]. Subsequent ADVANCE analyses similarly suggested greater coronary risk with HbA1c ≥ 8% among *Hp 2-2* participants, although the overall phenotype-by-HbA1c interaction was not significant [23]. Beyond glycemia, ACCORD Lipid and Blood Pressure analyses suggested possible HP phenotype–dependent variation in the relationships of HDL cholesterol, fenofibrate therapy, and intensive blood-pressure lowering with cardiovascular outcomes [36,37,38]. Mechanistic studies have additionally linked *Hp 2-2* to increased HDL-bound hemoglobin and impaired coronary endothelial function [39]. The FIELD study further indicated that circulating haptoglobin concentration and HP phenotype provide partly non-equivalent information regarding cardiovascular risk and treatment response [40], while the LURIC cohort did not demonstrate a direct association between *HP* genotype and mortality but suggested effect modification by vitamin E status, particularly among individuals with diabetes [41]. Collectively, these heterogeneous findings support a model of *HP 2–2* as a context-dependent susceptibility genotype whose clinical expression is influenced by glycemic exposure, vascular phenotype, antioxidant milieu, and treatment context rather than as a uniformly adverse cardiovascular genotype. This framework is particularly relevant to kidney transplantation, where diabetes, residual kidney dysfunction, hypertension, dyslipidemia, immunosuppressive exposure, and chronic oxidative-inflammatory stress frequently coexist; however, the present study lacked standardized longitudinal HbA1c and detailed treatment-exposure data and therefore cannot directly evaluate *HP* genotype × metabolic or therapeutic interactions. Consistent with these mechanistic observations, *HP 2–2* has been associated with altered HDL-related biology, including increased HDL-bound hemoglobin, impaired endothelial function, and abnormalities in apolipoprotein A1 and lecithin–cholesterol acyltransferase pathways [36,39,42,43]. This is relevant to the present cohort because LDL cholesterol, triglycerides, and total cholesterol were similar between genotype groups, suggesting that the observed cardiovascular and LVEF associations may relate more to HDL dysfunction and hemoglobin-mediated oxidative vascular injury than to conventional lipid burden alone.

The echocardiographic findings add a clinically relevant but exploratory dimension to the genotype–phenotype association. *HP 2–2* recipients had lower continuous LVEF, and the *HP2* allele-dosage analysis supported a graded inverse association with LVEF; however, *HP 2–2* was not independently associated with LVEF < 60%. The absolute difference was modest: the unadjusted median difference was 3 percentage points and the primary adjusted model estimated an approximately 2.0-percentage-point lower LVEF in *HP 2–2* recipients, while median LVEF remained within the conventionally preserved range in both groups. Preserved conventional LVEF does not exclude subclinical myocardial dysfunction in diabetes [44], but the present findings should be interpreted as an intermediate cardiac functional phenotype rather than evidence of overt systolic dysfunction.

The additional confounding sensitivity analyses further refine this interpretation. The association between *HP 2–2* and documented cardiovascular disease remained directionally similar after adjustment for individual transplant-related covariates but was no longer statistically significant in the fully expanded model, supporting a cautious, hypothesis-generating interpretation. By contrast, the continuous LVEF estimate was comparatively stable across broader adjustment and remained directionally consistent after additional adjustment for documented cardiovascular disease burden. Together with the concordant *HP2* allele-dosage association, this supports the stability of the observed LVEF signal without establishing causality. Prior reports linking HP-related phenotypes to vascular dysfunction, subclinical vascular disease, unstable plaque, coronary disease, and cardiovascular death provide a broader biological context for this intermediate cardiac phenotype [39,45,46,47,48,49,50,51].

The absence of an association between *HP* genotype and hard transplant-related outcomes is also clinically informative. Overall biopsy-proven rejection, progression to ESKD/graft failure, death-censored graft survival, and all-cause mortality were not significantly different between *HP 2–2* and non–*HP 2–2* recipients. This is consistent with the large Norwegian kidney transplant cohort reported by Dupont et al., in which *HP 2–2* was not significantly associated with cardiovascular mortality, all-cause mortality, or graft loss [30]. Hard outcomes are late, multifactorial endpoints shaped by competing immunologic, infectious, metabolic, hemodynamic, donor-related, and treatment-related factors and may therefore be relatively insensitive to genotype-related intermediate cardiovascular phenotypes, particularly in a single-center cohort with limited event numbers. The present findings do not contradict prior survival data; rather, they suggest that any HP-related signal in kidney transplantation may be more apparent in intermediate cardiometabolic or cardiac functional phenotypes than in graft failure or mortality. This interpretation is also consistent with broader transplant literature. Haptoglobin variation has been associated with outcomes in other transplant settings, including liver transplantation, cardiac allograft vasculopathy after heart transplantation, and inflammatory complications after hematopoietic cell transplantation [27,28,29]. However, the direction and clinical relevance of these associations likely depend on organ type, donor–recipient biology, oxidative injury, immune activation, and outcome definition. In kidney transplantation, graft failure and mortality are multifactorial endpoints; a single oxidative-stress-related genotype would therefore not be expected to explain hard transplant outcomes in isolation.

To our knowledge, this study is among the first kidney transplant cohorts to evaluate recipient *HP* genotype across cardiometabolic, echocardiographic, and transplant-related domains rather than focusing only on patient or graft survival. Recent studies have renewed interest in *HP* genotype as a modifier of cardiovascular risk, glycemic exposure, HDL function, and treatment response in diabetes and coronary disease [21,22,23,35,36,37,38,39,40,41,42]. In this context, kidney transplant recipients represent a biologically relevant population because they experience persistent cardiometabolic stress, chronic inflammation, immunosuppressive exposure, and substantial cardiovascular event burden despite restoration of kidney function. The present findings therefore provide a rationale for prospective studies integrating *HP* genotype with longitudinal glycemic profiles, oxidative-stress markers, HDL functional assays, protocolized echocardiography, and adjudicated cardiovascular outcomes.

A key strength of this study is its integrated phenotyping of cardiometabolic, laboratory, echocardiographic, and transplant-related outcomes within a single kidney transplant cohort. The a priori *HP 2–2* versus non–*HP 2–2* comparison was complemented by three-genotype and *HP2* allele-dosage analyses, while parsimonious multivariable models and additional transplant-related sensitivity analyses were used to evaluate robustness without overinterpreting event-limited models. Continuous LVEF was available across the full analytic cohort, death-censored graft survival was analyzed over the full available follow-up using Kaplan–Meier analysis and Cox regression, and FDR correction was applied to prespecified adjusted model families to distinguish nominal associations from signals retained after multiplicity correction.

This study has several limitations. It was retrospective, single-center, and restricted to clinically stable outpatient kidney transplant recipients. A particularly important limitation is the timing of echocardiography. Examinations were obtained during routine clinical care rather than at a prespecified post-transplant time point, and the selected studies therefore occurred at heterogeneous times after transplantation. The exact transplant-to-echocardiography interval was not reliably available and could not be incorporated into the multivariable models. Because cardiac structure and function may vary with post-transplant duration, graft function, treatment exposure, and intervening cardiovascular events, temporal confounding cannot be excluded; graft follow-up duration in the sensitivity analyses should not be interpreted as a surrogate for the transplant-to-echocardiography interval. Structural echocardiographic variables were additionally unavailable in 38 of 229 recipients, and availability differed by genotype and several measured characteristics, introducing potential differential referral, ascertainment, and selection bias. Continuous LVEF was available in all 229 recipients but remained subject to the non-uniform timing of routine-care echocardiography. Potential confounding by sex was addressed a priori: female sex was similarly distributed between *HP 2–2* and non–*HP 2–2* recipients (33.6% vs. 37.3%; *p* = 0.56), sex was included in all multivariable echocardiographic models, and left atrial enlargement was defined using sex-specific thresholds. Nevertheless, residual sex-related confounding cannot be completely excluded.

The cardiometabolic phenotypes also lacked detailed longitudinal characterization. Cardiovascular disease was defined as documented coronary artery disease, prior myocardial infarction, coronary revascularization, ischemic stroke, or peripheral artery disease, but subtype-specific timing relative to transplantation, severity, recurrence, and treatment were not uniformly available. Hypertension was captured as a documented clinical comorbidity, without uniformly available data on pre- versus post-transplant onset, duration, longitudinal blood-pressure control, severity, or treatment intensity. Diabetes mellitus combined pre-existing and post-transplant disease; timing of onset, disease duration, longitudinal HbA1c exposure, and detailed treatment trajectories were not uniformly available. These limitations constrain temporal and mechanistic interpretation and leave potential residual confounding despite multivariable and sensitivity analyses. Additional limitations include the modest number of hard outcome events; absence of systematic coronary imaging, strain imaging, protocolized serial echocardiography, cardiac biomarker profiling, and direct measures of oxidative stress, hemoglobin-scavenging capacity, or HDL function; lack of circulating haptoglobin concentration and donor *HP* genotype; absence of additional *HP*-locus characterization; and lack of an external validation cohort. Circulating haptoglobin is a dynamic acute-phase biomarker that is not interchangeable with genetically determined *HP* genotype [40,52], and donor haptoglobin variation may also be relevant in transplantation [27]. Accordingly, the present findings should be interpreted as exploratory genotype–phenotype associations rather than evidence of causality or independent clinical risk prediction.

## 5. Conclusions

In conclusion, recipient *HP 2–2* genotype showed nominal associations with greater documented cardiovascular disease burden and lower continuous LVEF in adult kidney transplant recipients, whereas no association was observed with overall biopsy-proven rejection, graft failure, death-censored graft survival, or all-cause mortality. In complementary allele-dosage analyses, increasing *HP2* allele burden showed a graded inverse association with LVEF that remained after FDR correction. Given the retrospective single-center design, temporal heterogeneity of phenotype ascertainment, potential structural-echocardiography selection bias, and residual confounding, these findings should be considered hypothesis-generating. Larger prospective multicenter studies with adjudicated cardiovascular outcomes, longitudinal metabolic phenotyping, protocolized echocardiography, and external validation are required before *HP* genotype can be considered for clinical risk stratification.

## Figures and Tables

**Figure 1:**
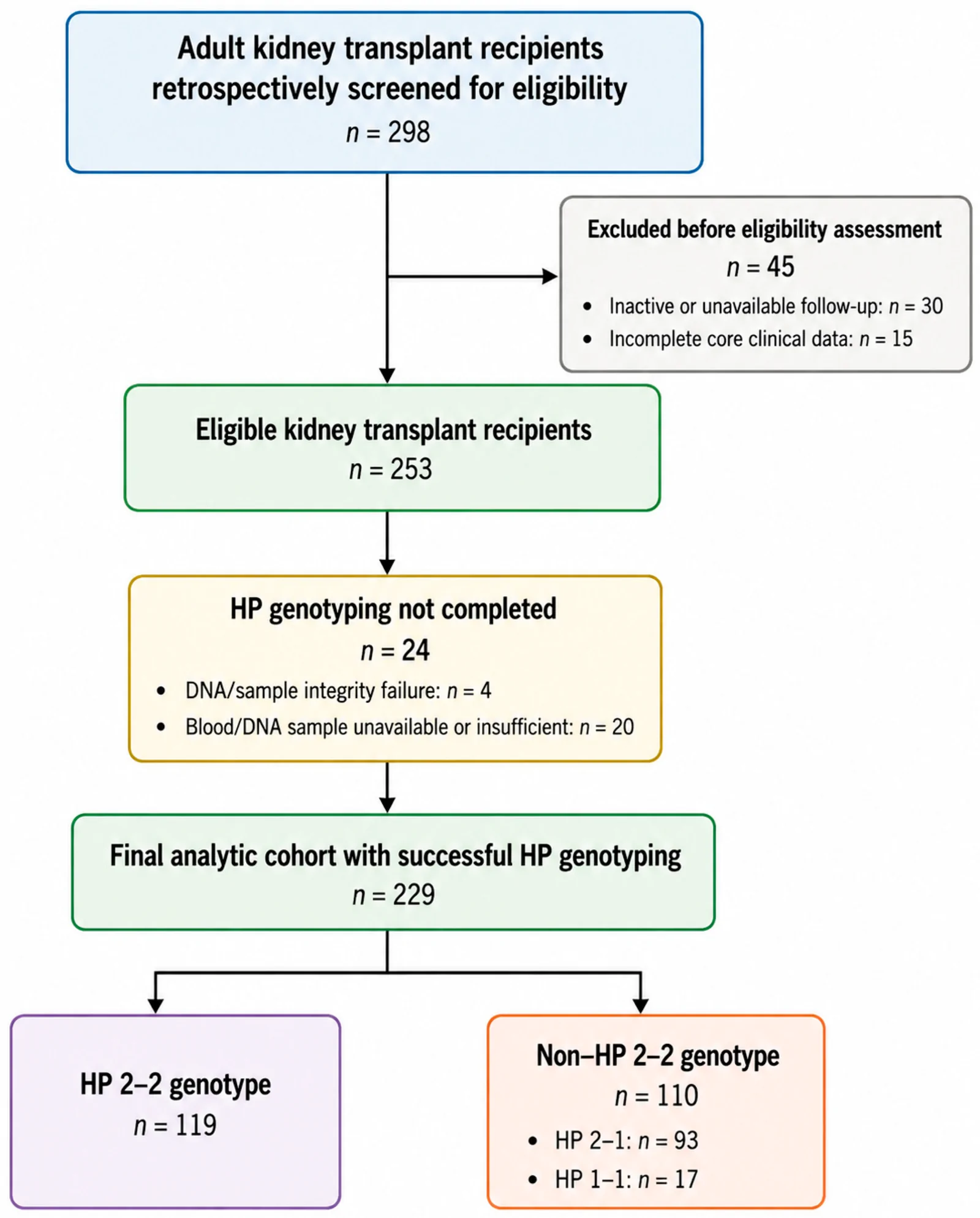
Study flow diagram. Flow diagram showing the selection of adult kidney transplant recipients included in the final analytic cohort. Among 298 screened recipients, 45 were excluded before eligibility assessment because of inactive or unavailable follow-up or incomplete core clinical data. Among 253 eligible recipients, *HP* genotyping was successfully completed in 229. The final cohort included 119 recipients with *HP 2–2* genotype and 110 recipients with non–*HP 2–2* genotype, comprising 93 *HP 2–1* and 17 *HP 1–1* recipients. HP, haptoglobin.

**Figure 2:**
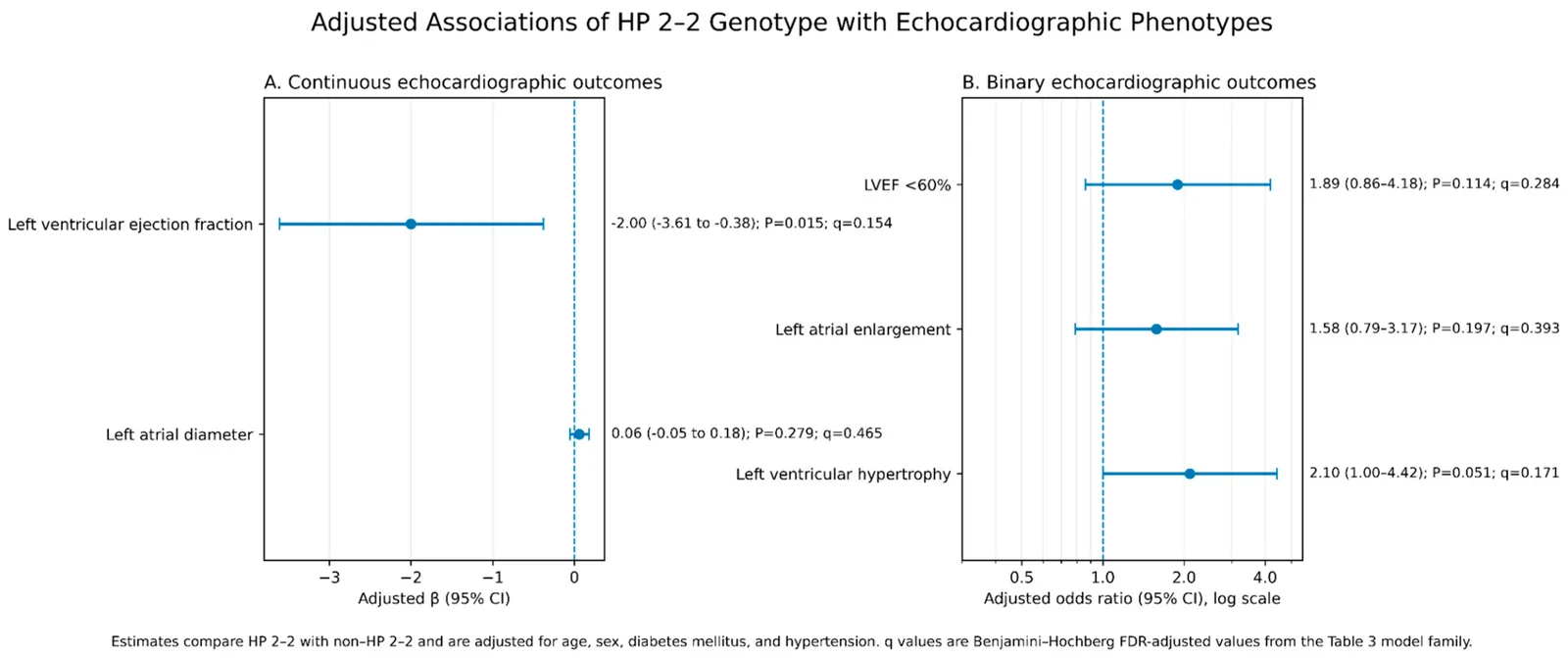
Adjusted associations of *HP 2–2* genotype with echocardiographic phenotypes. Forest plots show multivariable-adjusted associations comparing *HP 2–2* with non–*HP 2–2* recipients. Panel (**A**) presents adjusted β coefficients with 95% confidence intervals for continuous echocardiographic outcomes; β coefficients represent percentage-point differences for left ventricular ejection fraction and centimeter differences for left atrial diameter. Panel (**B**) presents adjusted odds ratios with 95% confidence intervals for binary echocardiographic outcomes, including LVEF < 60%, sex-defined left atrial enlargement, and left ventricular hypertrophy; odds ratios are displayed on a logarithmic scale. All models were adjusted for age, sex, diabetes mellitus, and hypertension. Points indicate adjusted effect estimates and horizontal lines indicate 95% confidence intervals. Vertical reference lines indicate no association (β = 0 for continuous outcomes and OR = 1 for binary outcomes). Nominal *p* values and Benjamini–Hochberg FDR-adjusted q values are shown; q values were calculated across the 10 adjusted outcome models reported in Table 3. CI, confidence interval; FDR, false discovery rate; HP, haptoglobin; LVEF, left ventricular ejection fraction; OR, odds ratio.

**Figure 3:**
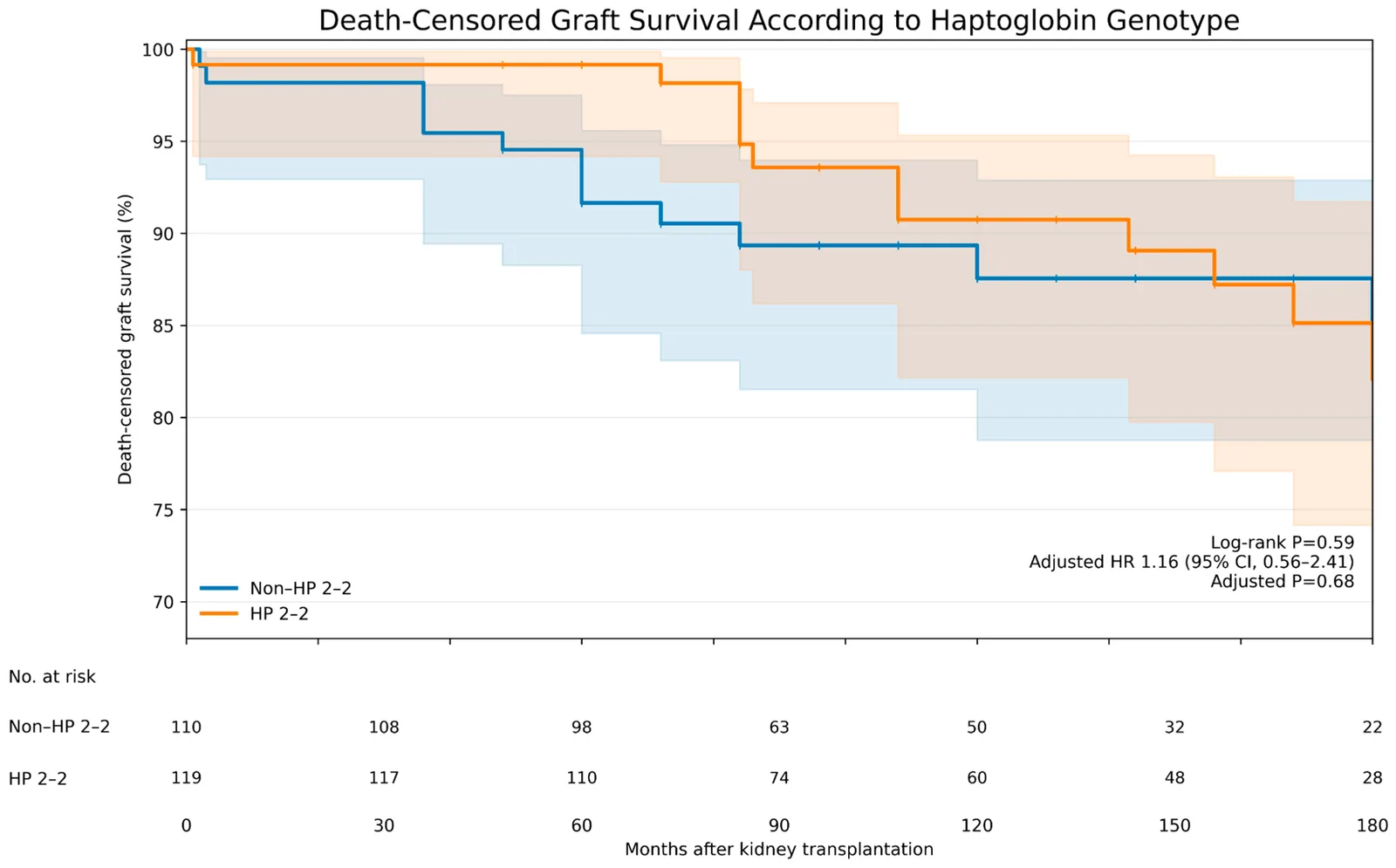
Death-censored graft survival according to haptoglobin genotype. Kaplan–Meier curves show death-censored graft survival in *HP 2–2* and non–*HP 2–2* recipients. Shaded areas, displayed in the same colors as the corresponding Kaplan–Meier curves, represent pointwise 95% confidence intervals, and tick marks indicate censored observations. Death with a functioning graft was treated as a censoring event. Numbers at risk are shown at 0, 30, 60, 90, 120, 150, and 180 months. Curves are displayed through 180 months for visualization because the number of recipients remaining at risk becomes limited thereafter; statistical analyses used the full available follow-up period. Survival distributions were compared using the log-rank test. The adjusted hazard ratio was estimated using Cox proportional hazards regression including *HP 2–2* genotype, age, sex, and donor type. CI, confidence interval; HP, haptoglobin; HR, hazard ratio.

**Table 1:** Baseline and Transplant-Related Characteristics of Kidney Transplant Recipients According to Haptoglobin Genotype.

Characteristic	Overall (*n* = 229)	*HP 2–2* (*n* = 119)	Non–*HP 2–2* (*n* = 110)	*p* Value
Age, yr, median (IQR)	41 (32–50)	41 (34–50)	38 (29–50)	0.13
Female sex, *n* (%)	81 (35.4)	40 (33.6)	41 (37.3)	0.56
**Donor type, *n* (%)**				0.06
Living donor	175 (76.4)	85 (71.4)	90 (81.8)	
Deceased donor	54 (23.6)	34 (28.6)	20 (18.2)	
**Dialysis modality before transplantation, *n* (%)**				0.79 *
Preemptive transplantation	28 (12.2)	17 (14.3)	11 (10.0)	
Hemodialysis	161 (70.3)	81 (68.1)	80 (72.7)	
Peritoneal dialysis	32 (14.0)	17 (14.3)	15 (13.6)	
Hemodialysis + peritoneal dialysis/other	8 (3.5)	4 (3.4)	4 (3.6)	
**Primary cause of kidney failure, *n* (%)**				0.39
Glomerulonephritis/immunologic diseases	68 (29.7)	37 (31.1)	31 (28.2)	
Diabetic nephropathy	22 (9.6)	14 (11.8)	8 (7.3)	
Other causes	139 (60.7)	68 (57.1)	71 (64.5)	
**Calcineurin inhibitor use, *n* (%)**				0.38
Tacrolimus	178 (77.7)	94 (79.0)	84 (76.4)	
Cyclosporine	30 (13.1)	17 (14.3)	13 (11.8)	
None	21 (9.2)	8 (6.7)	13 (11.8)	
mTOR inhibitor use, *n* (%)	30 (13.1)	12 (10.1)	18 (16.4)	0.16
Antimetabolite use, *n* (%)	202 (88.2)	106 (89.1)	96 (87.3)	0.67
**Maintenance steroid dose, *n* (%)**				0.34
None	12 (5.2)	7 (5.9)	5 (4.5)	
≤5 mg/day	200 (87.3)	106 (89.1)	94 (85.5)	
>5 mg/day	17 (7.4)	6 (5.0)	11 (10.0)	

Bold text identifies grouped categorical variables. * Fisher–Freeman–Halton exact test. HP, haptoglobin; IQR, interquartile range; mTOR, mechanistic target of rapamycin.

**Table 2:** Documented Cardiometabolic Phenotypes, Laboratory Measures, and Echocardiographic Characteristics According to Haptoglobin Genotype.

Characteristic	Overall (*n* = 229)	*HP 2–2* (*n* = 119)	Non–*HP 2–2* (*n* = 110)	*p* Value
**Documented cardiometabolic disease burden**				
Hypertension, *n* (%)	163 (71.2)	85 (71.4)	78 (70.9)	0.93
Diabetes mellitus, *n* (%)	42 (18.3)	28 (23.5)	14 (12.7)	0.03
Cardiovascular disease, *n* (%)	41 (17.9)	29 (24.4)	12 (10.9)	0.008
Malignancy, *n* (%)	25 (10.9)	11 (9.2)	14 (12.7)	0.40
**Post-transplant infection events**				
CMV infection, *n* (%)	29 (12.7)	16 (13.4)	13 (11.8)	0.71
BK viremia, *n* (%)	10 (4.4)	6 (5.0)	4 (3.6)	0.75 *
**Laboratory measures at last follow-up**				
Blood urea nitrogen, mg/dL, median (IQR)	20 (15–36)	19 (15–33)	22 (15–38)	0.20
Serum creatinine, mg/dL, median (IQR)	1.20 (0.99–1.99)	1.20 (1.02–1.98)	1.19 (0.99–2.00)	>0.99
Fasting plasma glucose, mg/dL, median (IQR)	91 (86–106)	90 (86–108)	91 (86–105)	0.74
LDL cholesterol, mg/dL, median (IQR)	116 (90–139)	114 (90–137)	118 (90–144)	0.77
Triglycerides, mg/dL, median (IQR)	134 (90–200)	136 (90–182)	126 (91–202)	0.90
Total cholesterol, mg/dL, median (IQR)	189 (153–227)	187 (159–226)	190 (152–232)	0.85
**Echocardiographic parameters**				
Left ventricular ejection fraction, %, median (IQR)	66 (60–69)	65 (60–67)	68 (62–70)	<0.001
Left ventricular ejection fraction < 60%, *n* (%)	33 (14.4)	22 (18.5)	11 (10.0)	0.07
Left atrial diameter, cm, median (IQR)	3.6 (3.3–4.0)	3.7 (3.3–4.0)	3.5 (3.3–3.8)	0.06
Left atrial enlargement, *n*/N (%)	61/191 (31.9)	41/107 (38.3)	20/84 (23.8)	0.03
Left ventricular hypertrophy, *n*/N (%)	60/191 (31.4)	43/107 (40.2)	17/84 (20.2)	0.003

(*) Fisher’s exact test was used for sparse two-category comparisons. Bold text identifies section headings. BK, BK polyomavirus; CMV, cytomegalovirus; HP, haptoglobin; IQR, interquartile range; LDL, low-density lipoprotein; Diabetes mellitus and cardiovascular disease represent cumulative documented clinical phenotypes recorded before transplantation or during post-transplant follow-up and should not be interpreted as baseline-only or incident outcomes. Echocardiographic analyses used available-case data; outcome-specific denominators are reported where applicable.

**Table 3:** Adjusted Associations of *HP 2–2* Genotype With Cardiovascular, Echocardiographic, and Transplant-Related Outcomes.

Outcome	Overall (*n* = 229)	*HP 2–2* (*n* = 119)	Non–*HP 2–2* (*n* = 110)	Unadjusted *p* Value	Adjusted Estimate for *HP 2–2*	Adjusted *p* Value	FDR q Value
Cardiovascular and echocardiographic outcomes							
Documented cardiovascular disease, *n* (%)	41 (17.9)	29 (24.4)	12 (10.9)	0.008	aOR 2.32 (95% CI, 1.07–5.03)	0.033	0.163
Left ventricular ejection fraction, %, median (IQR)	66 (60–69)	65 (60–67)	68 (62–70)	<0.001	β −2.00 (95% CI, −3.61 to −0.38)	0.015	0.154
Left ventricular ejection fraction < 60%, *n* (%)	33 (14.4)	22 (18.5)	11 (10.0)	0.07	aOR 1.89 (95% CI, 0.86–4.18)	0.114	0.284
Left atrial diameter, cm, median (IQR)	3.6 (3.3–4.0)	3.7 (3.3–4.0)	3.5 (3.3–3.8)	0.06	β 0.06 (95% CI, −0.05 to 0.18)	0.279	0.465
Left atrial enlargement, *n*/N (%)	61/191 (31.9)	41/107 (38.3)	20/84 (23.8)	0.03	aOR 1.58 (95% CI, 0.79–3.17)	0.197	0.393
Left ventricular hypertrophy, *n*/N (%)	60/191 (31.4)	43/107 (40.2)	17/84 (20.2)	0.003	aOR 2.10 (95% CI, 1.00–4.42)	0.051	0.171
Transplant-related outcomes							
Overall biopsy-proven rejection, *n* (%)	31 (13.5)	14 (11.8)	17 (15.5)	0.41	aOR 0.80 (95% CI, 0.37–1.74)	0.581	0.685
Progression to ESKD/graft failure, *n* (%)	31 (13.5)	18 (15.1)	13 (11.8)	0.46	aOR 1.27 (95% CI, 0.59–2.76)	0.543	0.685
Death-censored graft follow-up duration, mo, median (IQR)	108 (72–168)	120 (78–168)	102 (63–156)	0.15	—	—	—
Death-censored graft failure	31 events	18 events	13 events	0.59	aHR 1.16 (95% CI, 0.56–2.41)	0.685	0.685
All-cause mortality, *n* (%)	24 (10.5)	12 (10.1)	12 (10.9)	0.84	aOR 0.83 (95% CI, 0.33–2.05)	0.682	0.685

Notes: Adjusted estimates compare *HP 2–2* with non–*HP 2–2*. Cardiovascular disease and echocardiographic models were adjusted for age, sex, diabetes mellitus, and hypertension; rejection and graft-failure models were adjusted for age, sex, and donor type; all-cause mortality was adjusted for age and sex. Benjamini–Hochberg FDR correction was applied across the 10 adjusted outcome models shown in this table; the descriptive graft follow-up-duration comparison was excluded from the FDR family. Exact model-derived *p* values were used for FDR correction before rounding. aHR, adjusted hazard ratio; aOR, adjusted odds ratio; CI, confidence interval; ESKD, end-stage kidney disease; FDR, false discovery rate; HP, haptoglobin; IQR, interquartile range.

## Data Availability

Data supporting this study are available from the corresponding author on reasonable request, subject to ethics approval and data protection requirements.

## Supplementary Materials

The following supporting information can be downloaded at https://www.mdpi.com/article/10.3390/jcm15197536/s1, Supplementary File S1: Detailed GAP-PCR Primer Design and Haptoglobin Genotyping Methodology; Supplementary Figure S1: GAP-PCR design and representative electrophoretic patterns for haptoglobin genotyping; Supplementary Figure S2: Mechanistic comparison of haptoglobin genotypes and hemoglobin-scavenging efficiency; Supplementary Table S1: Clinical, Echocardiographic, and Transplant Outcomes According to Three Haptoglobin Genotypes; Supplementary Table S2: Adjusted *HP2* Allele-Dosage Models for Clinical, Echocardiographic, and Transplant Outcomes; Supplementary Table S3: Comparison of recipients with and without available structural echocardiographic data; Supplementary Table S4: Additional multivariable sensitivity analyses for residual confounding.

## Abbreviations

aHR, adjusted hazard ratio; AMR, antibody-mediated rejection; aOR, adjusted odds ratio; BK, BK polyomavirus; CAD, coronary artery disease; CI, confidence interval; CMV, cytomegalovirus; CNI, calcineurin inhibitor; CPRA, calculated panel reactive antibody; CVD, cardiovascular disease; DNA, deoxyribonucleic acid; DSA, donor-specific antibody; EDTA, ethylenediaminetetraacetic acid; eGFR, estimated glomerular filtration rate; ESKD, end-stage kidney disease; FDR, false discovery rate; HDL, high-density lipoprotein; HLA, human leukocyte antigen; HP, haptoglobin; *HP1*, haptoglobin 1 allele; *HP2*, haptoglobin 2 allele; HR, hazard ratio; HWE, Hardy–Weinberg equilibrium; IQR, interquartile range; LA, left atrial; LDL, low-density lipoprotein; LVEF, left ventricular ejection fraction; LVH, left ventricular hypertrophy; MFI, mean fluorescence intensity; MI, myocardial infarction; mTOR, mechanistic target of rapamycin; OR, odds ratio; PAD, peripheral artery disease; PCR, polymerase chain reaction; PRA, panel reactive antibody; TCMR, T cell–mediated rejection.
