# Supplementary material for: Association of Haptoglobin Genotype with Cardiovascular Disease and Left Ventricular Ejection Fraction in Kidney Transplant Recipients
Source: J Clin Med. 2026 Sep 28;15(19):7536. doi: 10.3390/jcm15197536 (PMC13666109; doi:10.3390/jcm15197536)
Supplement: Supplementary file 1 [file jcm-15-07536-s001.zip › jcm-4468339-supplementary.pdf]

Supplementary Materials

Association of Haptoglobin Genotype with Cardiovascular Disease and Left Ventricular Ejection Fraction in Kidney Transplant Recipients

Supplementary File S1. Detailed GAP-PCR Primer Design and Haptoglobin Genotyping Methodology

Three primers were used to selectively amplify the two haptoglobin alleles using a GAP-PCR approach: HP_2F (ACTAGCTTTCCACTCCTCCTTGTC), HP_GAP_F (CAGTTCGCGAGCTTCTATTCGG), and HP_6R (TCTAACAAAGGAAGTGGGAAATACTG). HP_2F and HP_GAP_F served as forward primers, whereas HP_6R served as the reverse primer.

For the HP1 allele, HP_GAP_F lacks a complementary target sequence and therefore does not contribute to amplification. Amplification with HP_2F and HP_6R generates a 2050-bp product.

For the HP2 allele, both the HP_2F–HP_6R and HP_GAP_F–HP_6R primer combinations are theoretically capable of amplification. Because the HP2 allele contains a duplicated sequence, amplification with HP_2F and HP_6R would generate a larger 3769-bp product, which is inefficiently amplified, if detectable at all. Consequently, the predominant diagnostic product for the HP2 allele is the 1769-bp amplicon generated by HP_GAP_F and HP_6R. In heterozygous samples, both the 2050-bp and 1769-bp products are observed.

Genomic DNA was extracted using the EZ1 Advanced system (QIAGEN, Germany). PCR amplification was performed using all three primers in each reaction. Amplification products were resolved by 2% agarose gel electrophoresis, and genotypes were assigned according to the resulting band pattern. A single 2050-bp band was classified as HP 1–1, a single 1769-bp band as HP 2–2, and the presence of both bands as HP 2–1. Representative electrophoretic banding patterns are shown in Supplementary Figure S1.

Supplementary Figure S1. GAP-PCR design and representative electrophoretic patterns for haptoglobin genotyping.

Figure S1A

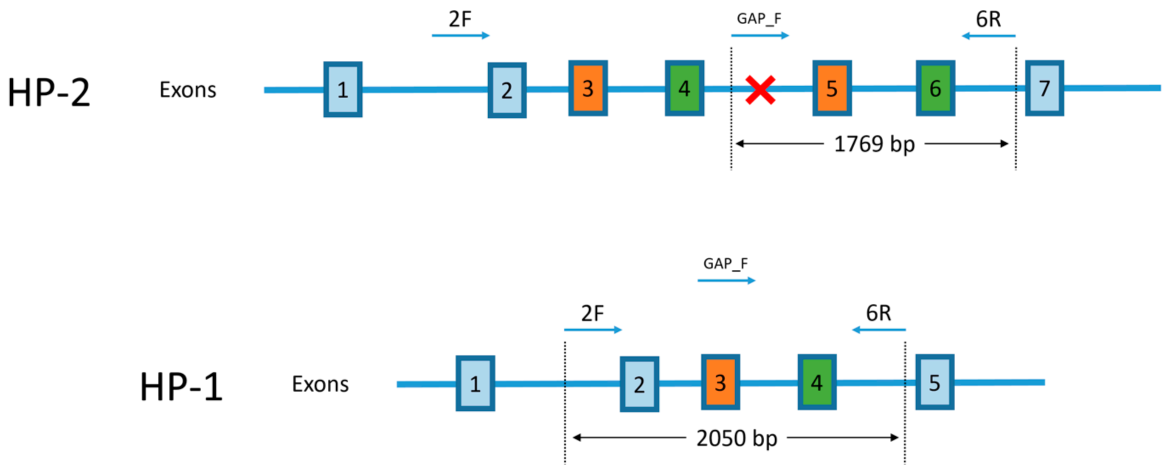

Figure S1B

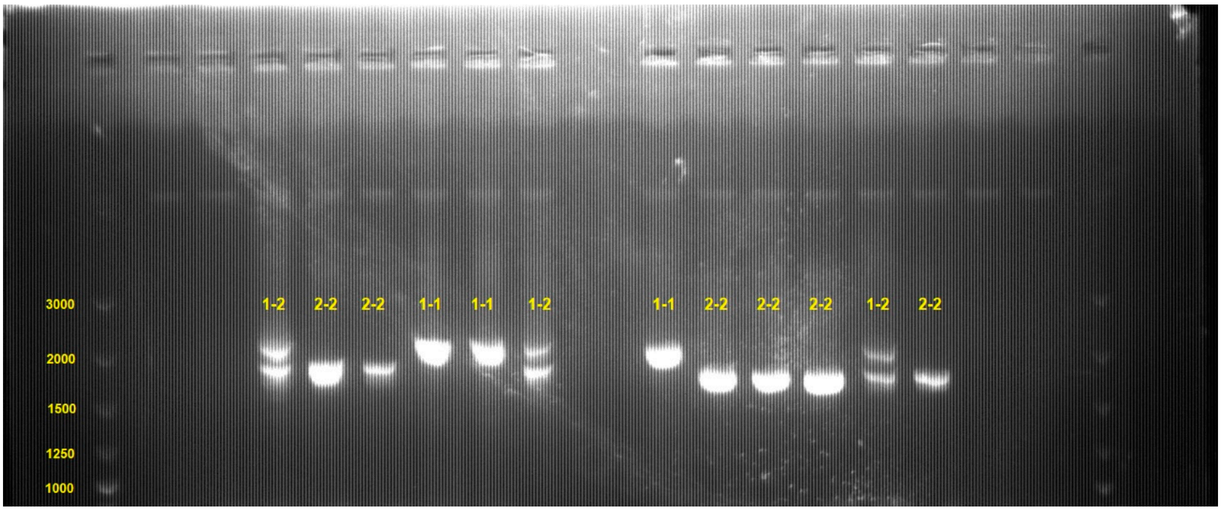

Supplementary Figure S1. GAP-PCR design and representative electrophoretic patterns for haptoglobin genotyping.

(A) Schematic representation of the GAP-PCR design used to distinguish the HP1 and HP2 alleles. The positions of the HP_2F, HP_GAP_F, and HP_6R primers and the diagnostically relevant amplicon sizes are shown. Amplification of the HP1 allele generates a 2050-bp product, whereas amplification of the HP2 allele generates a diagnostically informative 1769-bp product. The schematic is not drawn to scale.

(B) Representative agarose gel electrophoresis image showing the banding patterns used for genotype assignment. A single 2050-bp band was classified as HP 1–1, a single 1769-bp band as HP 2–2, and the

presence of both bands as HP 2–1. Molecular size markers are shown at the margins, and genotype calls are indicated above the corresponding lanes.

Abbreviations: HP, haptoglobin; PCR, polymerase chain reaction; bp, base pairs.

Supplementary Figure S2

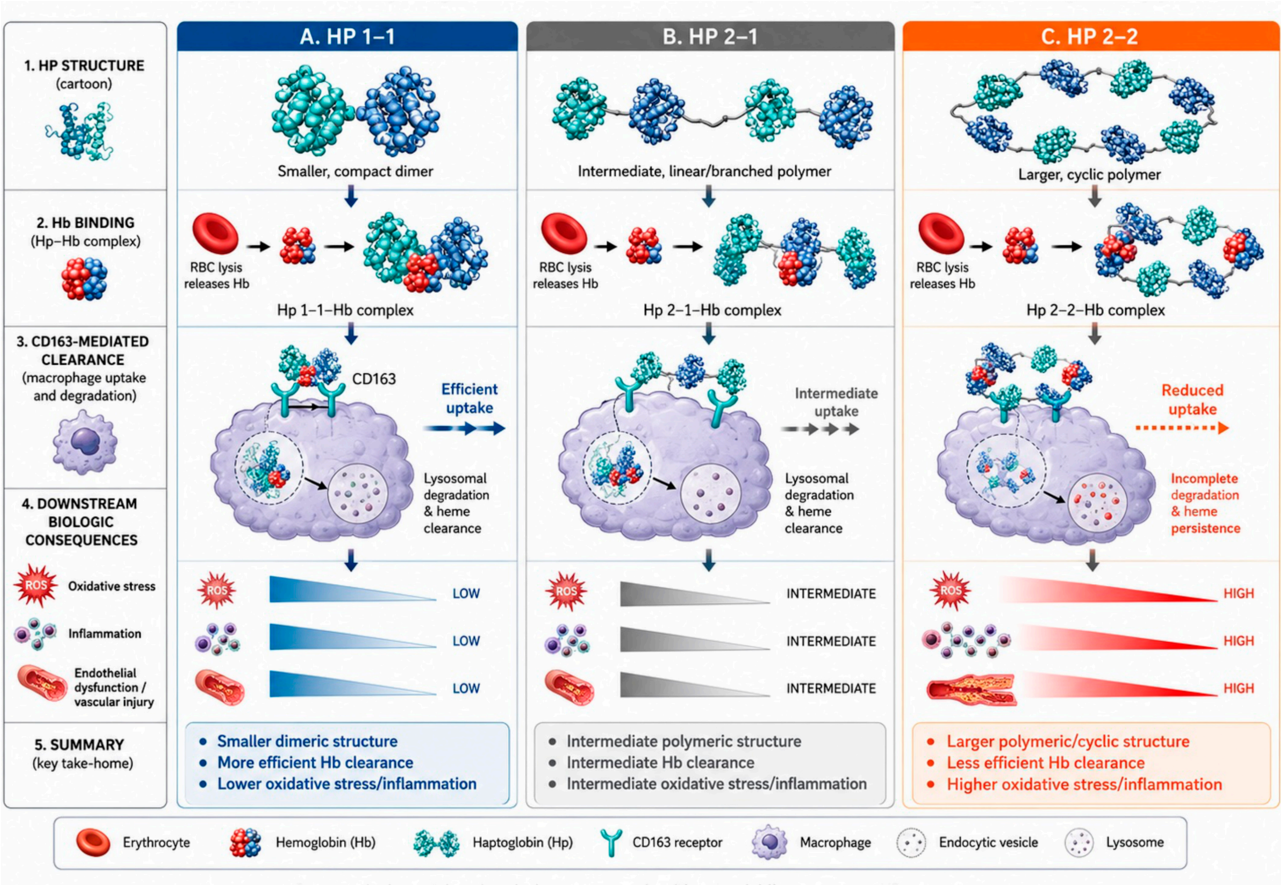

Supplementary Figure S2. Mechanistic comparison of haptoglobin genotypes and hemoglobin-scavenging efficiency. Conceptual schematic illustrating the structural and functional differences among HP 1–1, HP 2–1, and HP 2–2 genotypes. HP 1–1 is represented as a smaller, compact dimeric structure associated with more efficient hemoglobin binding, CD163-mediated macrophage uptake, lysosomal degradation, and heme clearance. HP 2–1 is shown as an intermediate polymeric structure with intermediate hemoglobin-scavenging efficiency. HP 2–2 is represented as a larger polymeric/cyclic structure associated with less efficient hemoglobin–heme complex clearance, greater persistence of redox-active heme burden, and higher downstream oxidative stress, inflammation, endothelial dysfunction, and vascular injury. The schematic is intended as a conceptual visual summary of known genotype-dependent structural and functional differences and does not represent exact crystallographic molecular structures. Abbreviations: Hb, hemoglobin; HP, haptoglobin; RBC, red blood cell; ROS, reactive oxygen species.

Supplementary Table S1. Clinical, Echocardiographic, and Transplant Outcomes According to Three Haptoglobin Genotypes

Characteristic	HP 1–1 (n = 17)	HP 2–1 (n = 93)	HP 2–2 (n = 119)	p value
Age, yr, median (IQR)	41 (34–56)	38 (28–49)	41 (34–50)	0.21
Female sex, n (%)	6 (35.3)	35 (37.6)	40 (33.6)	0.81
Deceased donor, n (%)	5 (29.4)	15 (16.1)	34 (28.6)	0.08
Hypertension, n (%)	8 (47.1)	70 (75.3)	85 (71.4)	0.07
Diabetes mellitus, n (%)	0 (0.0)	14 (15.1)	28 (23.5)	0.03
Cardiovascular disease, n (%)	1 (5.9)	11 (11.8)	29 (24.4)	0.02
Left ventricular ejection fraction, %, median (IQR)	70 (67–70)	67 (62–70)	65 (60–67)	<0.001
Left ventricular ejection fraction <60%, n (%)	1 (5.9)	10 (10.8)	22 (18.5)	0.22
Left atrial diameter, cm, median (IQR)	3.6 (3.5–3.7)	3.5 (3.3–3.8)	3.7 (3.3–4.0)	0.15
Left atrial enlargement, n/N (%)	2/13 (15.4)	18/71 (25.4)	41/107 (38.3)	0.09
Left ventricular hypertrophy, n/N (%)	4/13 (30.8)	13/71 (18.3)	43/107 (40.2)	0.005
Overall biopsy-proven rejection, n (%)	2 (11.8)	15 (16.1)	14 (11.8)	0.63
Progression to ESKD/graft failure, n (%)	2 (11.8)	11 (11.8)	18 (15.1)	0.87
All-cause mortality, n (%)	2 (11.8)	10 (10.8)	12 (10.1)	0.94
Death-censored graft follow-up duration, mo, median (IQR)	144 (96–192)	96 (60–156)	120 (78–168)	0.08

Notes: Continuous variables are presented as median (interquartile range), and categorical variables as n (%) or n/N (%), as appropriate. Three-genotype analyses were considered descriptive because the HP 1–1 subgroup was small. Abbreviations: ESKD, end-stage kidney disease; HP, haptoglobin; IQR, interquartile range.

Supplementary Table S2. Adjusted HP2 Allele-Dosage Models for Clinical, Echocardiographic, and Transplant Outcomes

Outcome	Events/N or N	Adjusted covariates	Adjusted estimate per HP2 allele	p value	FDR q value
Diabetes mellitus	42/229	Age, sex	aOR 2.15 (95% CI, 1.15–4.03)	0.017	0.095
Cardiovascular disease	41/229	Age, sex, diabetes mellitus, hypertension	aOR 2.10 (95% CI, 1.05–4.18)	0.035	0.130
Left ventricular ejection fraction, %	229	Age, sex, diabetes mellitus, hypertension	β −1.93 (95% CI, −3.20 to −0.65)	0.0033	0.036
Left ventricular ejection fraction <60%	33/229	Age, sex, diabetes mellitus, hypertension	aOR 1.81 (95% CI, 0.93–3.55)	0.08	0.23
Left atrial diameter, cm	191	Age, sex, diabetes mellitus, hypertension	β 0.04 (95% CI, −0.05 to 0.14)	0.37	0.59
Left atrial enlargement	61/191	Age, sex, diabetes mellitus, hypertension	aOR 1.56 (95% CI, 0.87–2.78)	0.13	0.24
Left ventricular hypertrophy	60/191	Age, sex, diabetes mellitus, hypertension	aOR 1.63 (95% CI, 0.89–2.99)	0.11	0.24
Overall biopsy-proven rejection	31/229	Age, sex, donor type	aOR 0.89 (95% CI, 0.49–1.63)	0.71	0.74
Progression to ESKD/graft failure	31/229	Age, sex, donor type	aOR 1.21 (95% CI, 0.65–2.25)	0.56	0.74
All-cause mortality	24/229	Age, sex	aOR 0.89 (95% CI, 0.45–1.77)	0.74	0.74
Death-censored graft failure	31/229	Age, sex, donor type	aHR 1.14 (95% CI, 0.64–2.04)	0.66	0.74

Notes: HP2 allele dosage was coded as 0 for HP 1–1, 1 for HP 2–1, and 2 for HP 2–2; adjusted estimates are reported per additional HP2 allele. Abbreviations: aHR, adjusted hazard ratio; aOR, adjusted odds ratio; CI, confidence interval; ESKD, end-stage kidney disease; FDR, false discovery rate; HP, haptoglobin.

Supplementary Table S3. Comparison of Recipients With and Without Available Structural Echocardiographic Data

Characteristic	Structural echo available (n = 191)	Structural echo unavailable (n = 38)	|SMD|	p value	Test
HP 2–2 genotype	107/191 (56.0%)	12/38 (31.6%)	0.508	0.007	Fisher exact
Female sex	71/191 (37.2%)	10/38 (26.3%)	0.235	0.265	Fisher exact
Diabetes mellitus	38/191 (19.9%)	4/38 (10.5%)	0.263	0.250	Fisher exact
Hypertension	136/191 (71.2%)	27/38 (71.1%)	0.003	1.000	Fisher exact
Documented cardiovascular disease burden	38/191 (19.9%)	3/38 (7.9%)	0.352	0.104	Fisher exact
Deceased donor	45/191 (23.6%)	9/38 (23.7%)	0.003	1.000	Fisher exact
Age, yr	41 (32–52)	38 (32–45)	0.290	0.178	Mann–Whitney U
Serum creatinine, mg/dL	1.20 (1.00–2.08)	1.17 (0.97–1.40)	0.190	0.479	Mann–Whitney U
Graft follow-up duration, mo	108 (72–168)	114 (84–165)	0.002	0.624	Mann–Whitney U
**Dialysis modality before transplantation**			**0.405**	**0.397**	**Fisher–Freeman–Halton exact**
Preemptive	24/191 (12.6%)	4/38 (10.5%)	0.064		
Hemodialysis	130/191 (68.1%)	31/38 (81.6%)	0.315		
Peritoneal dialysis	29/191 (15.2%)	3/38 (7.9%)	0.230		
HD + PD/other	8/191 (4.2%)	0/38 (0.0%)	0.296		
**Primary cause of kidney failure**			**0.406**	**0.141**	**Fisher–Freeman–Halton exact**
Glomerulonephritis/immunologic	59/191 (30.9%)	9/38 (23.7%)	0.162		
Diabetic nephropathy	21/191 (11.0%)	1/38 (2.6%)	0.337		
Other causes	111/191 (58.1%)	28/38 (73.7%)	0.333		

Supplementary Table S3 (continued).

Characteristic	Structural echo available (n = 191)	Structural echo unavailable (n = 38)	|SMD|	p value	Test
**Calcineurin inhibitor regimen**			**0.251**	**0.264**	**Fisher–Freeman–Halton exact**
Tacrolimus	151/191 (79.1%)	27/38 (71.1%)	0.186		
Cyclosporine	25/191 (13.1%)	5/38 (13.2%)	0.002		
None	15/191 (7.9%)	6/38 (15.8%)	0.248		
**Maintenance steroid dose**			**0.479**	**0.106**	**Fisher–Freeman–Halton exact**
None	11/191 (5.8%)	1/38 (2.6%)	0.156		
≤5 mg/day	163/191 (85.3%)	37/38 (97.4%)	0.438		
>5 mg/day	17/191 (8.9%)	0/38 (0.0%)	0.442		
mTOR inhibitor use	22/191 (11.5%)	8/38 (21.1%)	0.260	0.119	Fisher exact
Antimetabolite use	169/191 (88.5%)	33/38 (86.8%)	0.050	0.784	Fisher exact

Notes: Continuous variables are presented as median (interquartile range), and categorical variables as n/N (%). Absolute standardized differences (SMDs) were used as the primary measure of between-group balance. Although continuous variables are summarized using medians and interquartile ranges, their SMDs were calculated from differences in means standardized by the square root of the average within-group variance; for binary variables, SMDs were calculated from differences in proportions standardized by the corresponding pooled variance. For multicategory variables, the overall SMD represents the generalized Mahalanobis standardized difference across K−1 indicator variables, with category-specific binary SMDs additionally reported. For descriptive interpretation, an SMD ≥0.10 was considered indicative of potentially meaningful imbalance. p values are provided as secondary descriptive measures and were calculated using the Mann–Whitney U test for continuous variables, Fisher's exact test for binary categorical variables, and the Fisher–Freeman–Halton exact test for multicategory variables. No multiplicity adjustment was applied because these comparisons were undertaken to characterize patterns of structural echocardiographic data availability and potential selection bias rather than to test prespecified study hypotheses. Structural echocardiographic availability was defined by the availability of left atrial diameter and left ventricular hypertrophy data; continuous LVEF was available for all 229 recipients. Serum creatinine was used as the kidney-function measure because it was directly recorded and complete in the locked dataset. Graft follow-up duration was included as the available longitudinal transplant-related measure; the exact interval from transplantation to echocardiography was not available.

Supplementary Table S4. Additional Multivariable Sensitivity Analyses for Residual Confounding

Sensitivity model	Additional covariate(s) beyond age, sex, diabetes mellitus, and hypertension	CVD: aOR for HP 2–2 (95% CI)	p value	LVEF: β for HP 2–2, percentage points (95% CI)	p value
Serum creatinine	Most recent serum creatinine	2.55 (1.15–5.65)	0.022	−2.06 (−3.68 to −0.44)	0.013
Graft follow-up duration	Graft follow-up duration, months	2.25 (1.03–4.89)	0.041	−1.97 (−3.60 to −0.35)	0.018
Donor type	Living vs. deceased donor	2.12 (0.97–4.66)	0.061	−2.04 (−3.68 to −0.41)	0.014
Dialysis modality	Preemptive, HD, PD, HD/PD-other	2.48 (1.13–5.42)	0.023	−2.03 (−3.65 to −0.41)	0.014
CNI regimen	Tacrolimus, cyclosporine, no CNI	2.39 (1.09–5.23)	0.029	−1.90 (−3.51 to −0.28)	0.022
mTOR inhibitor	mTOR inhibitor use	2.34 (1.08–5.08)	0.032	−1.85 (−3.46 to −0.23)	0.025
Antimetabolite	Antimetabolite use	2.32 (1.07–5.03)	0.033	−1.97 (−3.57 to −0.37)	0.016
Steroid category	None, ≤5 mg/day, >5 mg/day	2.26 (1.04–4.92)	0.039	−2.14 (−3.76 to −0.52)	0.010
Core transplant model	Creatinine + graft follow-up + donor type	2.20 (0.98–4.96)	0.057	−2.08 (−3.73 to −0.43)	0.014
Immunosuppression model	CNI + mTOR inhibitor + antimetabolite + steroid category	2.25 (1.02–4.93)	0.044	−2.02 (−3.63 to −0.41)	0.014
Fully expanded available-covariate model	Creatinine + graft follow-up + donor type + CNI + mTOR inhibitor + antimetabolite + steroid category	2.14 (0.94–4.86)	0.069	−2.00 (−3.65 to −0.36)	0.017
Fully expanded + documented CVD burden	Above + documented CVD burden	N/A	—	−1.72 (−3.36 to −0.09)	0.039

Complementary HP2 allele-dosage LVEF robustness: fully expanded available-covariate model β = −1.83 per additional HP2 allele (95% CI, −3.12 to −0.53; p = 0.006); fully expanded + documented CVD burden β = −1.60 (95% CI, −2.89 to −0.31; p = 0.015).

Notes: All models included age, sex, diabetes mellitus, and hypertension. Continuous LVEF was analyzed using multivariable linear regression and documented cardiovascular disease burden using multivariable logistic regression. Donor type was modeled as living versus deceased donor. Pre-transplant dialysis modality was modeled categorically. Calcineurin inhibitor regimen was modeled as tacrolimus, cyclosporine, or no calcineurin inhibitor; mTOR inhibitor and antimetabolite exposure were modeled as use versus no use; and maintenance steroid exposure was categorized as none, ≤5 mg/day, or >5 mg/day. The fully expanded cardiovascular disease model was considered exploratory because only 41 cardiovascular disease cases were available relative to model complexity. A separately adjudicated baseline-only cardiovascular disease variable, the exact transplant-to-echocardiography interval, validated dialysis duration, standardized longitudinal anemia/hemoglobin data, smoking status, and lipid-lowering treatment were not reliably available. p values for the nested sensitivity models are exploratory and were not subjected to an additional FDR correction because these models evaluate robustness of the same underlying associations rather than independent hypotheses. Abbreviations: aOR, adjusted odds ratio; CI, confidence interval; CNI, calcineurin inhibitor; CVD, cardiovascular disease; HD, hemodialysis; HP, haptoglobin; LVEF, left ventricular ejection fraction; mTOR, mechanistic target of rapamycin; PD, peritoneal dialysis.
